# Hypercholesterolaemia: optimal treatment by next-generation drugs?

**DOI:** 10.1007/s12471-015-0741-2

**Published:** 2015-09-01

**Authors:** E.E. van der Wall

**Affiliations:** Netherlands Society of Cardiology/Holland Heart House, Moreelsepark 1, 3511 EP Utrecht, The Netherlands

Statins are the current standard treatment of patients with hypercholesterolaemia. These drugs have been shown to be very effective in reducing the incidence of cardiovascular disease [1, 2]. Nevertheless, a substantial number of patients do not achieve their target low-density lipid protein cholesterol (LDL-C) levels. Consequently, new treatment options are required to decrease LDL-C levels beyond those currently achieved.

In July 2015 it was reported that Amgen Inc.’s cholesterol medication, evolocumab (Repatha), has received regulatory approval from the European Commission, making it the first regulatory clearance for a new class of powerful cholesterol-lowering drugs. Evolocumab is an inhibitor of PCSK9, which stands for protease proprotein convertase subtilisin/kexin 9 (PCSK9), an enzyme shown to down-regulate LDL-C receptor protein levels. Already more than 10 years ago, PCSK9 emerged as a validated target for lowering plasma LDL-C levels [3, 4]. PCSK9 inhibitors are designed to treat patients with persistently high LDL-C levels despite optimal use of statins. The drug has to be injected subcutaneously once every two weeks. The European Commission cleared evolocumab for patients with hazardously high cholesterol levels including those with inherited conditions. In the largest monotherapy trial using a PCSK9 inhibitor to date, evolocumab yielded significant LDL-C reductions compared with placebo or ezetimibe and was well tolerated in patients with hypercholesterolaemia [5]. Familial hypercholesterolaemia is an autosomal dominant genetic disorder, associated with elevated levels of LDL-C, which can lead to premature cardiovascular disease [6, 7]. In particular, homozygous autosomal dominant hypercholesterolaemia, an orphan disease caused by mutations in the LDL receptor, apolipoprotein B (APOB), or PCSK9, is characterised by elevated LDL-C levels [8].

At the same time as the approval of evolocumab, the US Food and Drug Administration (FDA) approved another PCSK9 inhibitor, alirocumab (Praluent) produced by Sanofi and Regeneron. Alirocumab was approved for people with an inherited condition that causes very high LDL-C levels, as well as for the millions of individuals who have sustained a myocardial infarction, cerebrovascular accident or other types of cardiovascular disease and whose LDL-C is higher than desirable [9]. Monotherapy with alirocumab demonstrated significantly greater LDL-C lowering versus ezetimibe after 24 weeks with the lower 75 mg dose sufficient to provide ≥ 50 % LDL-C reduction in the majority of the patients [10]. As PCSK9 is an attractive target for lowering plasma LDL-C with potential plaque-stabilising features, the ODYSSEY OUTCOMES trial was recently initiated to evaluate the efficacy of alirocumab in patients with cardiovascular disease [11]. This Phase 3 study will randomise approximately 18,000 patients to receive biweekly subcutaneous injections of alirocumab (75–150 mg) or matching placebo beginning 1 to 12 months after an index hospitalisation for acute myocardial infarction or unstable angina. ODYSSEY OUTCOMES will therefore determine whether the addition of the PCSK9 antibody alirocumab to intensive statin therapy reduces cardiovascular morbidity and mortality in patients with acute coronary syndromes [12]. As part of the overall ODYSSEY program, the European Heart Journal published the ODYSSEY COMBO II trial in May 2015, with the aim to compare the efficacy and safety of alirocumab, versus ezetimibe, as add-on therapy to maximally tolerated statin therapy in high cardiovascular risk patients with inadequately controlled hypercholesterolaemia [13]. In 720 patients it was shown that alirocumab achieved significantly greater reductions in LDL-C compared with ezetimibe, with a similar safety profile. The findings of the ODYSSEY COMBO II trial add to the evidence of alirocumab as a safe and efficacious option for patients whose LDL-C is insufficiently controlled under maximally dosed statin therapy, as is the case for an increasing number of high-risk patients.

The FDA is expected to have made a decision for evolocumab by the end of August 2015. Although PCSK9 inhibitors appear a valuable new option in the treatment of patients with persistently high LDL-C levels, the approval of either cholesterol drug by the FDA and the European Commission will fuel the combat over the high price of treatment by PCSK9 inhibitors. It has been estimated that the new drugs would cost US$ 7,000 to $ 12,000 a year (6380–10,940 €), which is significantly more expensive than existing generic statin alternatives. More recent data even suggest that the medication’s list price is approximately US$ 14,600 annually (12,760 €). For that reason, it is currently recommended that physicians limit prescribing only to the very-high-risk, hard-to-treat groups.

Of crucial importance is that more data are needed to establish whether the next-generation drugs actually translate into less cardiovascular morbidity and mortality. As a result, the definitive long-term results of the clinical trials that are in progress, such as the ODYSSEY OUTCOMES trial and the OSLER trials, have to be awaited [12, 14]. At present, statins are available as low-cost generics, are well tolerated in most patients, and their effectiveness is supported by strong evidence in a large spectrum of cardiovascular diseases [15–18]. In the meantime, physicians are advised to follow the current guidelines, which recommend lifestyle change and, if needed, statins for most patients with or at risk of heart disease. Improving diet and optimising exercise remain the cornerstones of heart disease management and prevention.

## Funding

None

## Conflict of interest

None declared
